# Loneliness during COVID-19: Development and influencing factors

**DOI:** 10.1371/journal.pone.0265900

**Published:** 2022-03-30

**Authors:** Charikleia Lampraki, Adar Hoffman, Angélique Roquet, Daniela S. Jopp

**Affiliations:** 1 Institute of Psychology, University of Lausanne, Lausanne, Vaud, Switzerland; 2 LIVES Centre, Swiss Centre of Expertise in Life Course Research, Lausanne, Switzerland; Charles Darwin University, AUSTRALIA

## Abstract

In early pandemic waves, when vaccination against COVID-19 was not yet an option, distancing and reduced social contact were the most effective measures to slow down the pandemic. Changes in frequency and forms of social contact have reduced the spread of the COVID-19 virus and thus saved lives, yet there is increasing evidence for negative side effects such as mental health issues. In the present study, we investigate the development of loneliness and its predictors to examine the role of changes in social networks due to social distancing and other COVID-19-related life changes. A total of 737 participants (age range = 18–81 years) completed an online survey in three waves during the last quarter of 2020 at one-month intervals. Latent growth and multilevel modeling revealed that emotional loneliness increased over time, while social loneliness remained stable. Moreover, socially lonely individuals were likely to also develop emotional loneliness over time. Increased social distancing and sanitary measures were accompanied by decreased social interactions and loss of individuals considered SOS contacts and confidants. Changes in specific social network indicators were differentially associated with changes in emotional vs social loneliness: Loss of friends considered confidants was associated with increasing emotional loneliness, whereas loss of friends considered SOS contacts and reduced overall social interactions were related to increasing social loneliness. Lastly, individuals with more family-and-friend SOS contacts, more friends as confidants and an overall higher number of social interactions were more protected from feeling socially or emotionally lonely. Study findings enhance the understanding of underlying mechanisms differentially contributing to social and emotional loneliness and offer practical suggestions to reduce mental-health side effects of social distancing.

## Introduction

Governments around the globe have tried to slow the spreading of the COVID-19 pandemic through strict public health measures, including lockdowns and “stay-at-home” orders. In the absence of vaccination or effective medical treatment, social distancing (defined as individuals reducing physical contact with people outside their household [1]), along with increased hand hygiene and wearing masks, were considered the most effective tools to reduce infections and protect particularly vulnerable individuals at earlier stages of the pandemic. Implemented measures included curfews, quarantines, and closing of (non-essential) stores, schools, universities, and cultural and sports locations to reduce contact in public. These measures included many country-specific rules limiting social contact in private (e.g., number of people and/or households allowed to meet). In Switzerland, in contrast to other European countries, there was no lockdown during the second wave of the pandemic (November-December 2020); instead, regional governments only issued recommendations and specific measures to be followed due to increased COVID-19 infection cases (i.e., no restrictions in October; closing of non-essential shops, cultural events, and sports, recommendation to work from home in November; closing of restaurants and, if feasible, obligatory home-office work in December). While strict social distancing and sanitary measures were effective to limit new infections in many countries (e.g., Italy) [2], there is increasing evidence that these measures had immediate and unprecedented consequences for individuals’ psycho-social functioning. Studies have shown that social distancing orders and lockdowns lead to considerable disruption in people’s behaviors and daily habits, leading to social isolation and negative consequences for psychological well-being and mental health [3–5]. Consequently, the Swiss government’s approach of choosing a “semi-lockdown” and issuing recommendations, relying more strongly on citizen’s personal responsibility, rather than externally enforcing social distancing through strict lockdown measures, represents an interesting sample case to investigate the extent to which such milder measures have mental health consequences.

So far, there are few longitudinal studies able to cast light on the underlying mechanisms responsible for worsening well-being and mental health (e.g., [6–8]). While an increasing number of studies provide evidence for short-term mental health risks associated with the ongoing pandemic and hint to potentially long-term consequences (e.g., depression [9,10]; anxiety [11,12]; addictive behaviors [13,14]; suicidal ideation [6,7]), these studies mostly used cross-sectional data collected during the first pandemic wave of COVID-19. The goal of the present study was to illustrate how the advancement of the pandemic during the second pandemic wave, together with increasingly restrictive social distancing orders, led to quantitative and qualitative changes in social networks and how these affected loneliness, using a large longitudinal dataset. Besides identifying risks and protective factors that can be addressed through adjustments of measures and recommendations during the ongoing COVID-19 pandemic, the present work also offers insights on basic mechanisms underlying the development of loneliness.

### Loneliness and its predictors

Loneliness refers to an emotional state in which individuals experience a feeling of isolation, detachment and lack of social support and belonginess [15]. Loneliness represents an important mental health risk factor that has long been ignored; meanwhile, there is substantial evidence showing that its negative consequences are comparable to smoking and obesity (e.g., [16]). Lonely individuals also have a 50% (up to 80% for chronically lonely) higher all-cause mortality rate than individuals with healthy social relations [17]. During the COVID-19 pandemic, loneliness has become a concern quickly, as a consequence of imposed social distancing, lockdown, and home office orders: Being confined at home reduced habitual (e.g., at work) and casual (e.g., in the street) in-person interactions, and led to a loss of face-to-face communication with significant others with whom individuals did not share the same household (e.g., older parents, adult children, friends [18,19]). Various studies have documented high levels of loneliness [6,20,21] and an increase in loneliness since the start of the pandemic [22,23].

Cross-sectional studies have shown that the experience of loneliness during the COVID-19 pandemic is associated with several risk factors, including sociodemographic, contextual, and social factors. Concerning demographic factors, younger age, being a woman, low household income, and being a student were identified as risk factors for higher loneliness (e.g., [18–21,24]). Among the contextual and social factors found to be protective against loneliness were living with others, residing in a rural area, having three or more close friends, and reporting high social support (e.g., [20,25,26]). Yet, a notable gap in the literature is the inclusion of other potential aspects of influence, specific to the ongoing COVID-19 crisis. Although dominating everyday life during the pandemic, little is known about the role of COVID-19-specific measures, such as the influence of sanitary and social-distancing measures, on feelings of loneliness [27]. Similarly, COVID-19-specific adaptation of communication habits (e.g., more phone calls or use of modern technology) may impact levels of loneliness, as suggested by a handful of studies. For example, Macdonald and Hülür [28] found that although older adults had fewer social interactions, those able to maintain satisfactory communication levels during the pandemic were protected against loneliness. While alternative communication means have been booming during the pandemic as safe options to replace actual face-to-face interactions, they do not necessarily have the same positive effects on well-being and mental health as “real” in-person interactions (e.g., [29,30]). For example, investigating the experience of the pandemic in four countries, Geirdal and colleagues [30] found that frequent users of social media had worse mental health, quality of life, and felt lonelier compared to less frequent social media users. More virtual social contact (e.g., via video conferencing, phone, or text messages) during the pandemic has also been found to be associated with higher loneliness [31]. These studies suggest that virtual in-person social exchanges may be better than no exchange, but these still seem to miss aspects that meeting others in person may offer.

While cross-sectional studies offer a snapshot of aspects associated with feeling more or less lonely, longitudinal studies provide information about potential mechanisms underlying the increase or decrease of loneliness over time; publication of such longitudinal study findings is still scarce, but become increasingly important with the temporal extension of the COVID-19 pandemic. Using a Spanish life-span sample assessed during the first pandemic wave, Losada-Baltar and colleagues [22] found that being younger was associated with more loneliness at the time of the lockdown. In addition, having more negative perceptions of aging, thinking of oneself as a burden, and expressing fewer positive emotions and more psychological distress were associated with more loneliness. On the other hand, individuals felt less lonely when they lived with others and had more contact with relatives, when they expressed themselves more through emotions, had better sleep quality, and more resources to entertain themselves.

### Social *vs* emotional loneliness

To gain a more fine-grained picture of mechanisms underlying loneliness, a closer look at loneliness may be useful, particularly when interested in the predictive value of COVID-19-specific measures, including changes in social resources due to social distancing. Loneliness is often treated as a unidimensional construct with different aspects of social relationships relating to its development, such as quantity or quality of the social network [32,33]. Yet, theory and empirical evidence suggest two underlying dimensions: social loneliness and emotional loneliness (e.g., [34,35]). According to Weiss [36], social loneliness results from the unmet need for social peer relationships and is thus experienced by people who are poorly socially integrated, whereas emotional loneliness results from unmet needs for close, intimate, or emotional contact with (available) significant others, such as one’s partner, parents, or children. Several empirical studies have found evidence supporting the bidimensionality of loneliness and differential predictors for both loneliness dimensions (e.g., [37–39]). For example, Green and colleagues [26] found that social and emotional loneliness were moderately correlated with each other, but they had different correlates: Social loneliness was more strongly correlated with items that assessed feelings of belongingness to a group of friends, while emotional loneliness was more strongly correlated with items that assessed feelings of closeness to others. In line with these findings, Diehl and colleagues [40] found in a sample of college students that higher social loneliness was present if students were immigrants, physically inactive (i.e., less engaged in college sports activities), and studying social sciences, whereas lower levels of emotional loneliness were associated with being married or in a committed relationship. In sum, these studies suggest that emotional and social loneliness are linked yet distinct facets of loneliness associated to various types of social deficits. Thus, social vs emotional loneliness could be affected differently by the COVID-19 pandemic: Specifically, being unable to meet others should increase social loneliness, whereas reduced interactions with emotionally valued social partners should increase emotional loneliness. In line with this assumption, Tilburg and colleagues [23] found that older Dutch individuals felt more lonely during lockdown (assessed in May 2020) compared to before the pandemic (assessed in October 2019), and that the increase was higher for emotional (moderate effect size: *d* = .49) compared to social loneliness (small effect size: *d* = .21). Being younger and living with a spouse/partner were associated with both lower social and emotional loneliness; unmet support needs and feeling affected by reduced outdoor activities were associated with higher social and emotional loneliness. Differential predictive patterns were also found: Being female and having more frequent contact with grandchildren was related to less social loneliness, while being female and reporting fewer social contacts and activities were related to more emotional loneliness. These findings show the usefulness of investigating social and emotional loneliness separately to increase the prediction of interindividual differences and their underlying mechanisms.

### The present study

The main goal of the present study was to investigate social and emotional loneliness and their predictors longitudinally over the course of the pandemic, namely during the development of the second pandemic wave (October to December 2020) within a life-span sample. To our knowledge, no previous research has investigated the longitudinal stability and interdependence of the two dimensions of loneliness (i.e., emotional and social loneliness) during the COVID-19 crisis. Specifically, we investigated how social and emotional loneliness developed and influenced each other over time while COVID-19 restrictions became increasingly severe. Based on the underlying theory [36,39,41], we expected that the experience of social loneliness would be related, yet distinct from emotional loneliness. As the COVID-19 crisis represents a unique and unprecedented context affecting individuals around the globe, we felt that studying social and emotional loneliness in this quasi-experimental setting, with short-term changes in potential predictors due to the aggravation of the pandemic, could provide important insights into the dynamic underlying mechanisms.

Following prior work on loneliness, we included the following sociodemographic and background variables as potential predictors: age, gender, time, marital status, living conditions, education, financial adequacy, employment, and student status as well as subjective health. Specifically, we expected that younger individuals were more likely to experience emotional loneliness, as the governmental recommendations restricted social exchanges that are central during young adulthood in finding or maintaining a close and meaningful connection with significant others outside one’s family [41]. In line with earlier studies [26,35], we expected that females would be more likely to experience emotional loneliness, but less likely to experience social loneliness. We further assumed that higher education, being a student, and working were associated with higher social and emotional loneliness, as these individuals likely experienced more disruptions due to COVID-19 measures (e.g., home-office requirements, online studies). We also assumed that more financial means and better health would be associated with less emotional loneliness (e.g., less stressful experience of the pandemic due to larger homes, better health insurance, or more means of communication).

Besides considering basic sociodemographic and contextual aspects, we were most interested in status and changes of diverse social contact indicators. Various COVID-19-specific measures (e.g., compliance with social distancing) and potential compensations (e.g., use of video conferencing, social media) were investigated as influencing factors of social and emotional loneliness. In line with theory and prior work [22,23], we expected that the course of the pandemic (i.e., increase of infections) and social distancing recommendations (in Switzerland, there was no lockdown during the second wave of the pandemic) led to a reduction of social contacts, which should result in an increase in social loneliness. Stay-home recommendations were assumed, at the same time, to also lead to a reduction of other important contacts from outside the household/primary family, such as confidants or individuals available to help if needed, and contact with loved ones, which in turn would lead to an increase in emotional loneliness. We also expected that the use of online means of communication would relate to feeling less socially and emotionally lonely, and that compliance with social distancing and sanitary measures would be associated with feeling lonelier.

## Materials and methods

### Sample and procedures

We conducted a longitudinal study on mental health consequences of COVID-19 in Switzerland during the second wave of the 2020 pandemic. We invited university students as well as people they knew (family members, friends, acquaintances) to participate in the study. There were no explicit inclusion criteria other than being older than 18 (legal age of majority in Switzerland) and mastery of French language (as the study was conducted in the French-speaking part of Switzerland). Exclusion criteria were not agreeing to informed consent. Participants filled out an online questionnaire which was administered at three time points (October, November, and December), allowing us to investigate status, change of loneliness, and potential predictors over the course of the three-month time period the study covered, during which the social and sanitary restrictions imposed and suggested by the Swiss government increased in severity to abate the spread of the coronavirus. Participants were aged 18 to 81 years old (*N* = 737, *M*_age_ = 31.58). Half of the participants were university students, and the other half from the general population, recruited through the students’ social network. The study was approved by the social science ethics commission of the University of Lausanne. Participants provided informed consent before being able to proceed to filling out the questionnaire.

### Measures

#### Sociodemographic and background variables

Demographic variables included *age*, *sex* (0 = *men*, 1 = *women*), *marital status* (*married*; 0 = *no*, 1 = *yes*), *living alone* (0 = *no*, 1 = *yes*), *education years*, *financial adequacy* (1 = *I do not have enough money to meet my needs* to 3 = *I have more than enough money to meet my needs*), being a *student* (0 = *no*, 1 = *yes*), and being *employed* (0 = *no*, 1 = *yes*). These variables had been assessed in the first study wave. *Time* represented the study wave and ranged from 0 = *study wave one (October)* to 2 = *study wave three (December)*. *Subjective health* was measured at every wave with a single question (“How do you evaluate your actual health?”). Participants answered on 5-point scale ranging from 1 = *very bad* to 5 = *very good*.

#### COVID-19-specific measures

*Sanitary measures* included two questions asking whether participants kept 1.5 meters from other people and whether they wore masks on public transportation or when they could not keep their distance. *Social-distancing measures* were assessed with two questions asking whether individuals reduced their social contacts and avoided shaking hands or sharing kisses to welcome others. The answering format ranged from 1 = *no*, *I continue as always* to *3 = yes*, *all the time*. A mean composite score was created to represent sanitary measures and social distancing, respectively, for each study wave (e.g., for sanitary measures wave 3 Spearman-Brown’s *ρ* = .50 & for social distancing wave 3 Spearman-Brown’s *ρ* = .64).

#### Communication tools

*Social media* (Facebook, Twitter), *traditional communication* (telephone, emails, letters), and *video communication* (e.g., Skype, Zoom) were assessed in each study wave, with one question asking for the frequency with which participants used the respective means of communication during the second wave of the pandemic (0 = *not used* to 4 = *more than three times per day*).

#### Social interactions indicators

*Social contacts* were measured with the six items from the short Lubben Social Network Scale (LSNS; [42]). The items assessed the *number of relatives in contact* (“With how many family members did you have contact during the past month?”) as *confidants* (“With how many family members can you talk about private matters?”) and as *SOS contacts* (“How many family members can you ask for help?”). The same items were also used, replacing “family members” with “friends” to identify the *number of friends in contact* as *confidants* and as *SOS contacts*. The answering format ranged from 0 = *nobody* to 5 = *nine or more people*. All items were assessed in every study wave. Even though the items were used as independent variables, a reliability analysis was conducted for the three family-related items and the three friends-related items indicating excellent reliability for the two constructs (e.g., family-related social contacts wave 3 Cronbach’s *α* = .81 and friends-related social contacts wave 3 Cronbach’s *α* = .84).

*Frequency of interactions* represents a mean-composite score constructed from three single questions asking participants whether they had interactions with *family*, *friends*, and *colleagues* during the past four weeks (e.g., wave 3 Cronbach’s *α* = .47). The answering format ranged from 0 = *no contact* to 4 = *yes*, *many times per day*. The questions were assessed in every study wave, and the time frame of four weeks was intended to reflect interactions since the last study wave.

#### Lonelines

*Social and emotional loneliness* were measured with the short De Jong Gierveld loneliness scale [43]. Three items assessed each dimension in every study wave (social loneliness: “There are plenty of people with whom I feel closely connected”; emotional loneliness: “I feel a general emptiness”). The answering format included 1 = *no*, *2 = mostly no*, *3 = more or less*, *4 = mostly yes*, and 5 = *yes*. Mean composite scores were built for social and emotional loneliness, respectively, with higher values indicating higher loneliness levels for each of them (e.g., social loneliness Cronbach’s *α* waves 1, 2, 3: .83, .82, .84; emotional loneliness Cronbach’s *α* waves 1, 2, 3: .77, .78, .80).

### Analytical strategy

We calculated descriptive statistics and correlations of study variables. In addition, we tested differences between study waves for variables that were assessed in every wave with a repeated-measures ANOVA.

For the main analysis, we used first a bivariate latent growth-curve model (LGM) to investigate the longitudinal associations between social and emotional loneliness, which was then followed by multilevel linear models with social and emotional loneliness as dependent variables to investigate predictive patterns. While LGM offers the advantage of modelling the structure and growth of multiple variables simultaneously, the adoption of a multilevel modelling approach allows for simpler model specification when including the level and change of multiple variables for the prediction of a single outcome. This two-step approach of testing a bivariate latent growth model followed by a separate multilevel model for each loneliness dimension, allows to combine the benefits of these two powerful statistical models.

As for the bivariate LGM analysis, we estimated the linear growth of social and emotional loneliness as well as all covariances between the slopes and the intercepts of both constructs. We allowed the variances of the intercept and slope to be estimated freely, with significant variances indicating that participants’ changes of social and emotional loneliness varied over time and differed around the initial level at wave 1. For each of the two dimensions of loneliness, we fitted linear slope loadings of [0, 1, 2] for waves 1 to 3 accordingly, and intercept loadings of [1, 1,1] [44]. Missing data were estimated with the Lavaan built-in FIML function, allowing us to use all available information (*N* = 737). No significant differences were found between the final model and a model implementing listwise deletion using only complete data (*n* = 504). LGM analysis were conducted in R [45], using the Lavaan package for structural equation modelling [46].

To address predictors of both loneliness constructs, we tested two multilevel linear models with social and emotional loneliness as dependent variables and background sociodemographic aspects (e.g., age, sex) and subjective health, COVID-19-specific measures (i.e., sanitary and social-distancing measures), communication tools (i.e., social media, traditional and video communication), and social network indicators (e.g., number of social contacts and frequency of interactions) as independent variables. We centered independent variables to enhance the interpretability of the within-subject results and obtain more stable estimates [47,48]. Person-mean-centered variables (i.e., subjective health, number of relatives in contact with, relatives being confidants, relatives being SOS contacts, and number of friends in contact with, friends being confidants, friends being SOS contacts, and frequency of interactions) were included in the models to test within-subject variation. To investigate between-subject differences, we included the person-mean of all variables in the model. Categorical variables (i.e., gender, married, living alone, student, employed) were not centered and entered into the models as factors. Random effects were also included in the models. We present the models that had the best fit to the data for each dependent variable, which we determined by two relative model fit indices, namely Akaike’s information criterion (AIC) and -2 log likelihood (-2LL). Although we tested within-subject change and between-subject differences for all the variables assessed repeatedly, for some, the inclusion of the respective person-mean-centered parameter (i.e., the parameter testing such changes) did not improve the model fit. Therefore, to obtain the most parsimonious model, we excluded them from the final models (i.e., the parameters indicating changes in social distancing and sanitary measures, changes in social media use, traditional and video communication). For each outcome, we calculated the intra-class correlation coefficient (ICC; [49]), first in fully unconditional models and then in every subsequent model to specify the amount of inter-individual and intra-individual variance explained by the hierarchical clustering of the data. The models were tested with maximum likelihood estimation using SPSS version 26.

## Results

**Fig 1:**
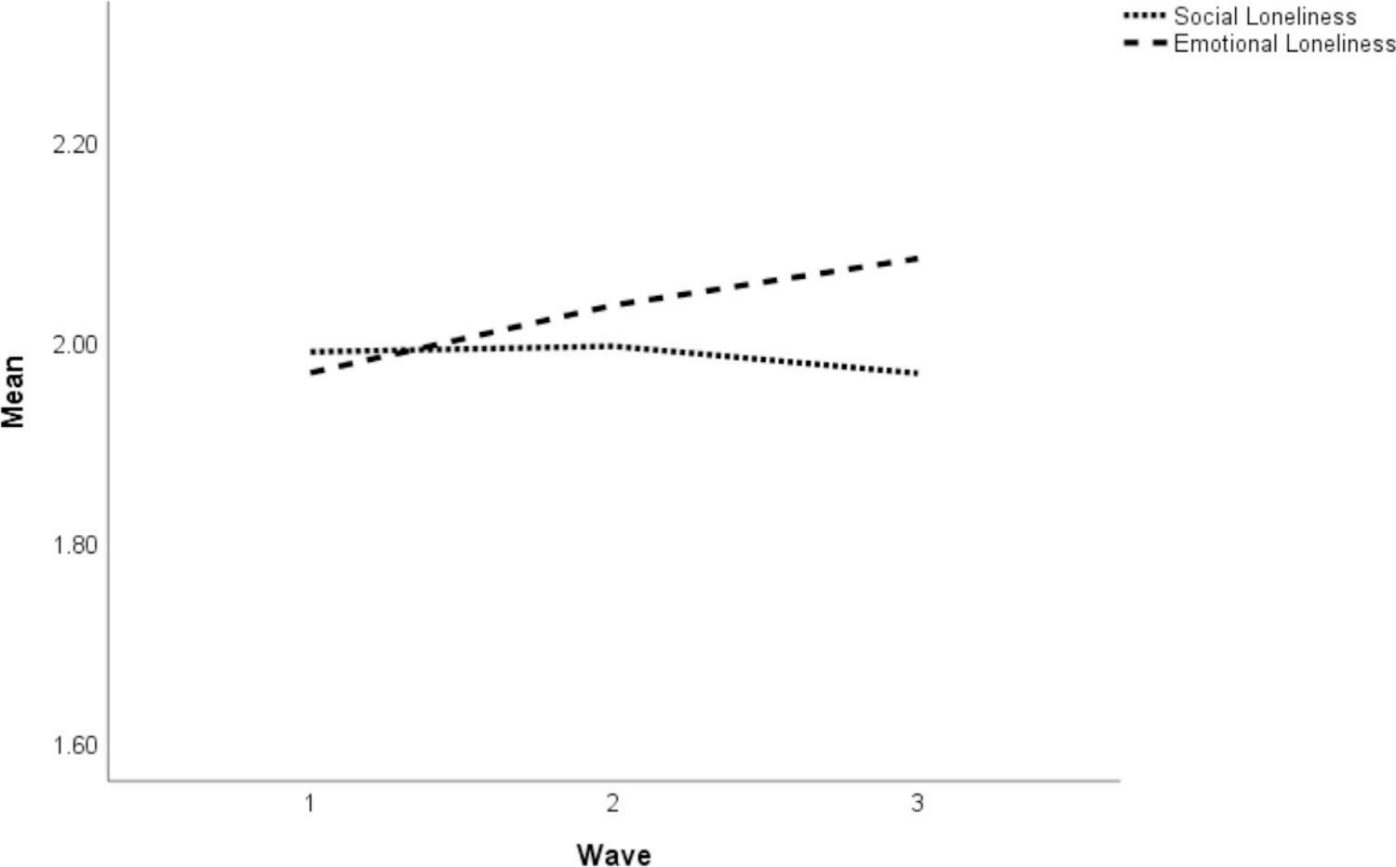
Mean levels of social and emotional loneliness across time.

Descriptive statistics and correlations are presented in Tables 1 and 2. We tested variables that changed over time for differences across study waves. These preliminary analyses indicated that over the course of the study, individuals increasingly followed sanitary and social-distancing measures, their subjective health got worse, social contacts became more limited, and the frequency of interactions decreased. In addition, we observed that emotional loneliness increased but social loneliness remained stable (Fig 1).

**Table 1:** Descriptive of study variables per study wave.

	Wave 1 (*N* = 737)	Wave 2 (*N* = 672)	Wave 3 (*N* = 539)	Difference Test
	*%* or *M* (*SD*)	*%* or *M* (*SD*)	*%* or *M* (*SD*)	*F*
Age	31.58 (13.80)	-	-	-
Sex (1 = women)	66.3	-	-	-
Married (1 = yes)	19.1	-	-	-
Living alone (1 = yes)	12.3	-	-	-
Education years	13.25 (2.73)	-	-	-
Financial adequacy	2.03 (0.62)	-	-	-
Student (1 = yes)	53.4	-	-	-
Employed (1 = yes)	37.2	-	-	-
Subjective health	4.11 (0.74)	4.04 (0.78)	3.98 (0.76)	6.53**
Social distancing measures	2.12 (0.58)	2.49 (0.54)	2.49 (0.54)	165.30***
Sanitary measures	2.48 (0.40)	2.64 (0.40)	2.61 (0.38)	42.26***
Social media	1.03 (0.96)	1.04 (0.98)	1.07 (0.98)	0.55
Traditional communication	2.83 (0.96)	2.80 (0.95)	2.83 (0.96)	0.53
Video communication	1.50 (1.45)	1.72 (1.44)	1.86 (1.48)	16.25***
N. relatives in contact	3.50 (1.01)	3.32 (0.95)	3.15 (1.04)	35.26***
N. relatives as confidant	2.34 (1.26)	2.32 (1.19)	2.30 (1.16)	0.13
N. relatives as SOS contact	2.68 (1.22)	2.64 (1.13)	2.54 (1.06)	7.22**
N. friends in contact	3.85 (1.15)	3.52 (1.20)	3.32 (1.22)	56.20***
N. friends as confidant	3.03 (1.21)	2.88 (1.19)	2.76 (1.17)	19.97***
N. friends as SOS contact	2.85 (1.17)	2.78 (1.18)	2.77 (1.16)	2.39^+^
Frequency of interactions	2.32 (0.73)	2.15 (0.77)	2.10 (0.77)	34.38***
Social loneliness	1.99 (0.91)	2.00 (0.91)	1.97 (0.91)	0.87
Emotional loneliness	1.97 (0.93)	2.03 (0.95)	2.08 (1.02)	6.59**

*Note*: N. = *Number of*.

^+^*p* < .10

*p < .05

**p < .01

***p < .001.

**Table 2:** Correlations between study variables at study wave 1 (*N* = 737).

	1	2	3	4	5	6	7	8	9	10	11	12	13	14	15	16	17	18	19	20	21	22	23
1. Age	1																						
2. Gender	-.10**	1																					
3. Married (1 = yes)	.64***	-.05	1																				
4. Living alone (1 = yes)	.01**	-.09*	-.18***	1																			
5. Education years	.30***	-.16***	.18***	.06	1																		
6. Financial Adequacy	.13**	-.16***	.16***	.01	.25***	1																	
7. Student (1 = yes)	-.61**	.20***	-.42***	-.08*	-.48***	-.22***	1																
8. Employed (1 = yes)	.43***	-.21***	.32***	.09*	.41***	.22***	-.83***	1															
9. Subjective health	-.06	-.13***	-.06	-.07^+^	.03	.18***	.05	.01	1														
10. Social distancing measures	.26***	.03	.25***	-.09*	.13**	.04	-.14***	.07^+^	-.10**	1													
11. Sanitary measures	.19***	.16***	.18***	-.03	.10**	-.02	-.06	.01	-.07^+^	.51***	1												
12. Social media	-.16***	-.02	-.17***	.04	-.06	-.03	.09*	-.06	-.01	-.07^+^	.001	1											
13. Traditional communication	.07^+^	-.05	.05	.07^+^^+^	.21***	.10**	-.10**	.17***	.04	.004	.02	.16***	1										
14. Video communication	-.26***	.06^+^	-.14***	-.05	-.01	-.03	.38***	-.29***	.08*	.02	-.003	.13**	.21***	1									
15. N. relatives in contact	.02	.08*	.07^+^	-.02	-.03	.11**	-.01	.03	.07^+^	-.01	.02	-.04	.07^+^	-.06	1								
16. N. relatives as confidant	.12**	.03	.11**	-.08*	.08*	.11**	-.12**	.14***	.11**	-.001	.04	-.02	.13***	-.06	.47***	1							
17. N. relatives as SOS contact	.14***	.03	.15***	-.09*	.04	.15***	-.11**	.12**	.18***	.01	.01	-.03	.11**	-.03	.47***	.65***	1						
18. N. friends in contact	-.37***	.10**	-.28***	.06	-.12**	.01	.30***	-.23***	.21***	-.22***	-.11**	-.04	.04	.08*	.26***	.14***	.10**	1					
19. N. friends as confidant	-.29***	.08*	-.26***	.03	.01	.05	.23***	-.12**	.15***	-.22***	-.15***	.04	.07^+^	.11**	.16***	.32***	.22***	.61***	1				
20. N. friends as SOS contact	-.18***	.08*	-.17***	-.06	.02	.07*	.14***	-.05	.21***	-.18***	-.05	.01	.12**	.12**	.21***	.35***	.42***	.50***	.70***	1			
21. Frequency of interactions	-.17***	.05	-.09*	-.001	-.03*	.10**	.11**	.06	.10**	-.12**	.0001	.01	.21***	.12**	.16***	.13***	.11**	.27***	.27***	.28***	1		
22. Emotional loneliness	-.11**	.06	-.12**	.14***	-.09^+^	-.14***	.10**	-.17***	-.36***	.08*	.02	.11**	-.10**	.08*	-.20***	-.29***	-.35***	-.16***	-.22***	-.35***	-.17***	1	
23. Social loneliness	.13**	-.06^+^	.10**	.08*	.07^+^	-.11**	-.10**	.02	-.26***	.14***	.05	.05	-.08*	-.01	-.21***	-.30***	-.33***	-.32***	-.43***	-.53***	-.28***	.56***	1

*Notes*: N. = *Number of*.

^+^*p* < .10

*p < .05

**p < .01

***p < .001.

### Longitudinal associations of social and emotional loneliness

**Fig 2:**
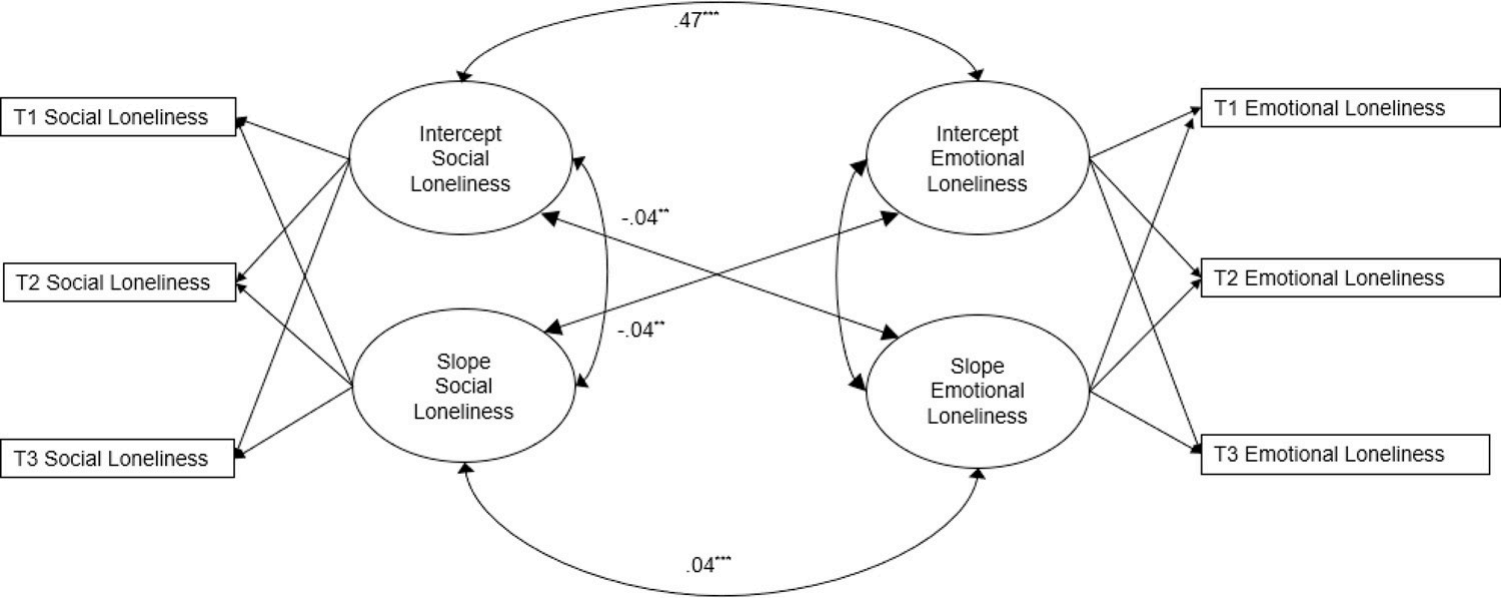
Cross-lagged correlations between intercepts and slopes of social and emotional loneliness in the bivariate LGM.

Table 3 presents the findings from the bivariate LGM, namely mean and variance parameters for the estimated latent variables, as well as intercept and slope correlations for social and emotional loneliness. The linear bivariate LGM showed a good fit to the data (*χ^2^ =* 27.10, *p* < .01, *df* = 11, *N* = 737, CFI = .99, TLI = .99, RMSEA = .045 (90% CI [.024, .066], SRMR = .015). The significant bivariate correlations between social and emotional loneliness presented in the final LGM model are depicted in Fig 2. We found a strong and positive intercept–intercept correlation, indicating that, as expected, when social loneliness was high, emotional loneliness was high as well. Moreover, results showed a significant negative correlation between the intercept of social loneliness and the slope of emotional loneliness, indicating that high social loneliness was associated with a smaller slope for emotional loneliness, reflecting a less steep increase in emotional loneliness when social loneliness was already high. We also found a significant negative correlation between the intercept of emotional loneliness and slope of social loneliness, suggesting that a higher level of emotional loneliness was associated with a smaller slope of social loneliness. Though the mean slope for social loneliness was estimated at zero, reflecting no significant mean change, the variance for the slope of social loneliness was significant (see Table 3), indicating that the slope for different participants varied around that mean. These findings show that individuals experienced social loneliness over time differently, with some feeling lonelier over time and others not experiencing any change. We also observed a moderate positive slope–slope correlation, indicating that social and emotional loneliness changed simultaneously over time: Those who experienced an increase in emotional loneliness were most likely to experience an increase in social loneliness, as well (despite the fact that no increase in social loneliness was observed for the total sample).

**Table 3:** Model parameters of the bivariate latent growth model of social and emotional loneliness.

	Intercept		Slope		Intercept–Slope Correlation
	*M*	*Variance*	*M*	*Variance*	*r*
Social loneliness	1.99***	.63***	-.001	.04***	-.03*
Emotional loneliness	1.97***	.62***	.06***	.05***	.004

Note

*p < .05

***p < .001.

### Factors influencing differences and change in social and emotional loneliness

We ran multilevel models to predict between-subject differences and within-subject changes in emotional and social loneliness. First, we estimated the within- and between-subject variance for social and emotional loneliness in separate, fully unconditional models. The ICC, which estimates the between-person variance as a proportion of the total variance, was *ρ* = .70 for social loneliness and *ρ* = .70 for emotional loneliness, indicating that individuals differed substantially from each other, allowing us to proceed to more complex multilevel models.

In the next steps of the analysis, we introduced groups of variables one at a time (i.e., demographics and subjective health, then COVID-19-specific measures, then communication means, finally social variables) to obtain the most parsimonious models, which are presented in Table 4. To increase readability, the effects of the background and central variables are presented in Table 4, excluding the non-significant effects of background variables (a table with the effects of all study variables is provided as supplementary material).

**Table 4:** Multilevel models with fixed and random effects of between- and within-subject covariates of emotional and social loneliness (*N* = 737).

	Social Loneliness		Emotional Loneliness	
	*B*	*SE*	*B*	*B*
*Fixed Between-Subject Effects*				
Age	.0002	.003	-.01**	.003
Living alone (0 = no)	-.07	.08	-.25**	.09
Subjective health	-.13**	.04	-.38***	.05
Education years	.03**	.01	.02	.01
Social distancing measures	.09	.07	.13	.08
Sanitary measures	-.12	.10	-.19	.11
Social media	.05*	.03	.11**	.03
Traditional communication	-.03	.03	-.06	.04
Video communication	.03	.02	.04	.03
N. relatives in contact	.04	.04	.02	.05
N. relatives as confidant	-.02	.04	.03	.04
N. relatives as SOS contact	-.14**	.04	-.19***	.05
N. friends in contact	-.01	.04	-.01	.04
N. friends as confidant	-.14**	.05	.06	.06
N. friends as SOS contact	-.26***	.05	-.24***	.05
Frequency of interactions	-.16***	.04	-.09^+^	.05
*Fixed Within-Subject Effects*				
Time	-.04*	.02	.03	.02
Subjective health	-.10**	.03	-.15***	.03
N. relatives in contact	-.04	.02	-.01	.02
N. relatives as confidant	-.04^+^	.03	-.03	.03
N. relatives as SOS contact	-.02	.03	-.02	.03
N. friends in contact	-.01	.02	-.002	.02
N. friends as confidant	.01	.03	-.06*	.03
N. friends as SOS contact	-.07**	.03	.02	.03
Frequency of interactions	-.09**	.03	-.04	.03
*Random Effects*				
Residual variance	.22***	.01	.23***	.01
Intercept	.27***	.02	.39***	.03
Slope (Frequency of interactions)	.03	.02	-	-
Slope (N. friends as confidant)	-	-	.05*	.02
Covariance intercept*slope	-.05*	.02	-.01	.02
-2 log likelihood (df)	3250.78 (35)		3536.73 (35)	
AIC	3320.78		3606.73	
ρ	.55		.63	

*Notes*: N. = *Number of*. df = *degrees of freedom*. AIC = *Akaike Information Criterion*. ρ = *Intraclass Correlation Coefficient*. Unstandardized estimates and standard errors are presented. Background variables with non-significant effects included (for details see extended table in appendix): Sex, Marital Status, Financial Adequacy, Student, Employed.

^+^*p* < .10

*p < .05

**p < .01

***p < .001.

#### Social loneliness

The between-subject effects in social loneliness showed that for the background variables, only education and health played roles: Higher education (*B* = .03; *p* < .01) and lower subjective health (*B* = -.13; *p* < .01) were associated with higher social loneliness. As for communication means, individuals who used more social media than the sample average experienced more social loneliness (*B* = .05, *p* < .05). Regarding social factors, individuals with a smaller number of relatives and friends as SOS contacts (compared to the sample average) experienced stronger feelings of social loneliness (relatives: *B* = -.14, *p* < .01; friends: *B* = -.26, *p* < .001). Additionally, individuals with a smaller number of friends serving as confidants (*B* = -.14, *p* < .01) and less frequent interactions than the sample mean (*B* = -.16, *p* < .001) experienced higher social loneliness.

Regarding the within-subject effects for social loneliness, a one unit decrease in subjective health was associated with an increase in social loneliness of *B* = -.10 (*p* < .01), indicating that health deterioration was related to feeling more socially lonely. In addition, time showed a significant negative effect (*B* = -.04, *p* < .05) indicating that individuals felt socially lonelier in earlier study waves than later; this negative trend did not reach significance levels in the SEM findings, and could be due to inclusion of predictor variables, clearing out some of the confounding variance in the MLM model. Furthermore, a decrease of one unit in the number of friends serving as SOS contacts was related to an increase of social loneliness equal to *B* = -.07 (*p* < .01), while a one-unit decrease in the frequency of interactions was associated with an increase of *B* = -.09 (*p* < .01) in social loneliness.

Considering the random effects, the within-subject random variance was significant (*B* = .22; *p* < .001), indicating that social loneliness varied significantly across time. In addition, the random intercept varied significantly (*B* = .27; *p* < .001), suggesting that the average level of social loneliness differed significantly across individuals. While the slope of frequency of interactions was not significant (*B* = .03; *p* > .05), indicating that the change in this variable was similar across participants, the covariance between this variable and the random intercept was significant (*B* = -.05; *p* < .05). This finding suggests that when a person had a high average level of social loneliness and their frequency of interactions increased, social loneliness decreased faster than for someone with a lower average level of social loneliness.

#### Emotional loneliness

For emotional loneliness, the between-subject effects showed that being younger (*B* = -.01; *p* < .01) was related to higher emotional loneliness. In addition, individuals who lived alone felt more emotionally lonely compared to those living with others (*B* = -.25; *p* < .01), and those with lower subjective health (*B* = -.38; *p* < .001) were more emotionally lonely. The latter two effects were relatively strong. Considering the social aspects, individuals who used more social media than the sample average experienced more emotional loneliness (*B* = .11, *p* < .01). In addition, having a larger number (than the sample mean) of relatives and friends as SOS contacts was associated with less emotional loneliness (relatives: *B* = -.19, *p* < .001; friends: *B* = -.24, *p* < .001). Similarly, individuals with more frequent interactions than the sample mean tended to feel less emotionally lonely (*B* = -.09, *p* < .10).

For the within-subject effects, a one-unit decrease in subjective health or in the number of friends serving as confidants was related to an increase of emotional loneliness (subjective health: *B* = -.15, *p* < .001; number of friends as confidants: *B* = -.06, *p* < .05). We also tested the random effects of the number of friends serving as confidants. The findings showed that this variable varied significantly across study waves between individuals (*B* = -.05; *p* < .05), indicating that the number of friends serving as confidants changed in a non-uniform way across participants. In addition, the random intercept varied significantly (*B* = .39; *p* < .001), indicating significant variability among individuals with respect to emotional loneliness. Within-subject random variance was also significant (*B* = .23; *p* < .001), suggesting that emotional loneliness varied across time with regard to each participant’s average level. The covariance between the random slope and the random intercept was not significant (*B* = .01; *p* > .05).

## Discussion

With this study, we aimed to investigate the development of social and emotional loneliness during the second COVID-19 pandemic wave, their longitudinal associations, and factors that influence them, within a life-span sample from the general population in Switzerland. The main findings show that compared to the beginning of the second wave of the COVID-19 pandemic in October 2020, individuals felt more emotionally lonely in November and December 2020. By contrast, social loneliness remained stable over the same time period when considering the total sample. We also found that when individuals felt very socially lonely, they had high levels of emotional loneliness at the same time. Our study further documented an increase in social distancing behaviors (e.g., less social contact), with negative consequences for individuals’ social networks (e.g., reports of less confidant and SOS contact). Finally, we found shared (e.g., frequency of interactions), but also differential predictors (e.g., age) of the level of social and emotional loneliness, as well as their change (e.g., number of friends as confidants).

Being compliant with governmental recommendations during the second wave of the COVID-19 pandemic, individuals increasingly engaged in sanitary and social-distancing measures. They significantly reduced their social contacts in terms of quantity: Face-to-face interactions with family, friends, and colleagues became less frequent. Modern communication tools, such as video conferencing, were used more frequently. However, we observed no compensatory increase in classic communication means, such as telephone or mail contacts. Of high importance is that at the same time, participants’ social networks changed in terms of quality: Participants lost social partners who could offer emotional support by being available to discuss personal matters (confidants) and practical support in times of need (SOS contacts). To the best of our knowledge, this is the first study showing not only quantitative but also qualitative changes in individuals’ social networks during the COVID-19 pandemic. This reduction in quantity and quality of social contact and networks increased significantly feelings of emotional and social loneliness, putting individuals in a more precarious position, as shown in the subsequent analysis.

### Emotional loneliness increased during the pandemic, but social loneliness remained stable

Even though social contacts and interactions became more limited due to governmental recommendations during the second wave of the pandemic, individuals did not feel more socially lonely. Instead, levels of emotional loneliness increased, confirming findings from a prior study conducted during the first pandemic wave [8]. The pandemic seems to have led to an enhanced need for closeness with significant others such as parents, romantic partners, or children, maybe due to the stress experienced in the face of the threat of the pandemic, resulting in increasing emotional loneliness given social-distancing orders. Seeking out close others in situations that trigger feelings of a limited future perspective, as suggested by the health and mortality dangers implied by COVID-19, is in line with Carstensen’s socioemotional selectivity theory (e.g., [50]), yet the pandemic also prompted fears of endangering those loved ones, which is a reason not to initiate close face-to-face contact. In line with this assumption, higher emotional loneliness went along with more experienced stress in a prior COVID-19 study [51]. We were able to observe how social and emotional loneliness influenced each other over time: For individuals who felt very socially lonely, emotional loneliness increased, but at a slower rate than for those who did not feel socially lonely, which may suggest that an upper limit was reached for both loneliness dimensions. These findings show not only that high emotional loneliness is related to high social loneliness for our sample, but also that the level of one dimension can influence the development of the other. Our study is one of the few that have examined the longitudinal stability and interdependence of social and emotional loneliness [39,41], and the only one, to our knowledge, that has investigated this relationship longitudinally during the pandemic. As the bidimensionality of loneliness has been demonstrated in many studies in the past (e.g., [26,39,52]), it is of great importance to better understand how the two dimensions interrelate and evolve over time to break the reinforcement of the vicious circle. Further investigation of their differential predictors may also help identify the drivers of the loneliness development over time and create targeted interventions for both loneliness types.

### Increased compliance with social-distancing and sanitary measures and its relation to social interactions and loneliness

In Switzerland, the second wave of the pandemic amplified the need for stricter compliance with the governmental recommendations at the beginning of November, along with other central-European countries [1]. In this study, we found that between the baseline assessment in October and the follow-ups in November and December, people adhered increasingly to social-distancing recommendations, such as reducing social contact and not shaking hands or welcome kisses, as well as the sanitary measures of wearing masks and keeping 1.5 meters of distance in public and/or crowded places. However, we did not find a direct link between increased compliance with the measures and increased social or emotional loneliness in our models, although the preliminary correlation analysis indicated that both facets of loneliness positively related to social-distancing measures. That these effects got lost when considering other social indicators suggests an underlying mediation effect: As people were more compliant with the measures, they reduced their social interactions and lost important specific partners (e.g., less SOS contact), which then increased their emotional loneliness. To our knowledge, only one study has tested the effect of compliance to social-distancing and sanitary measures, but on well-being, not loneliness [53]. Zhao and colleagues [53] reported that adoption, effectiveness, and perceived compliance with the measures was related to lower levels of anxiety, stress, and depressive symptoms. Thus, while compliance reduces stress and negative emotions, it seems to be indirectly increasing loneliness via loss of social interactions and important partners; such complex relationships should be investigated in more detail to gain a better understanding of the interplay among different behaviors, personal characteristics, and experiences during the pandemic. As compliance with social-distancing and sanitary measures did not explain any variance in loneliness in our study, in contrast to the Zhao and colleagues’ study [53], it is of interest to investigate the complexity of these relationships in more depth in the future.

### Lonely individuals use social media more often

The use of video communication had no impact on how lonely people felt; one could hypothesize that home-office orders led to more frequent video conferencing, but this did not necessarily increase contact with meaningful others, such as family members and friends. Similarly, the use of traditional means of communication, such as the telephone, was not linked to feeling less socially or emotionally lonely. These findings show that even though the phone was the most frequently used technical means to be in touch with family and friends in our study, its use did not increase during the second wave of the pandemic and was not related to either indicator of loneliness. This interpretation is opposed to previous studies, in which very old individuals were found to benefit from more telephone contact in terms of well-being, while younger old individuals did not, suggesting that talking to others on the phone did not compensate for in-person social contacts outside the context of mobility restrictions (health- or COVID-19-related) [54,55].

When people used social media more than average sample, they felt more socially and emotionally lonely. There could be a vicious circle: Lonely individuals may try to connect with others or get informed through social media, which does not help meet their need for closeness, leading to more loneliness. Such an interpretation is consistent with previous studies suggesting that social media use is negatively associated with feelings of loneliness and mental and psychosocial health (e.g., [29–31]). For example, Boursier and colleagues [29] reported that high feelings of loneliness predicted excessive social media use (e.g., Facebook, Instagram, Twitter), which was associated with increasing anxiety levels. This highlights that although social media can help one interact with others, its use can reinforce negative outcomes, including loneliness. In contrast to this previous research, the present study differentiates social media from traditional communication (such as the telephone) and video conferencing, offering further insights on which means of communication may help lonely individuals connect efficiently with others when in-person contact is not feasible.

### Lonely individuals had less contact with family and friends who could offer support

Emotionally and socially lonely individuals had fewer friends and relatives overall to whom they could turn to in times of need to get help. In addition, those who experienced social loneliness also had fewer friends to talk to about private issues. In line with previous research [56], these level differences show that lonely individuals are not only faced with a lower amount of contact but also with a poorer quality of these contacts.

Additionally, consistent with previous research reporting that contact quality may have a stronger impact on feelings of loneliness than contact quantity (e.g., [57]), our results can be explained by the fact that changes in the number of people one can ask for help or to talk to about private matters may be of great importance during the pandemic. Indeed, we found that a reduction in the number of friends to whom one can talk about private issues increased the feeling of emotional loneliness, while a reduction of the number of friends to whom one can turn for help increased social loneliness. These findings offer important insights, as they highlight that social networks have been negatively impacted during the second wave of the pandemic and that these changes had substantial consequences; one can hope that reactivating the relationships that suffered after the actual crisis will lead to reestablishing higher quality networks. Still, it may be important to stress that an active effort may be needed to make this happen, as has proven successful in prior loneliness interventions (e.g., [58]). Public health initiatives and mental health professionals may offer guidance to avoid these actual feelings of loneliness becoming chronic issues in the future.

### Less frequent interactions related to more social loneliness

Having overall less frequent social interactions was associated with stronger feelings of emotional (marginally) and social loneliness, and a decline in these interactions was related to increased social loneliness. These findings are in line with the theory assuming that quantity of social contacts should be linked to social loneliness [36]. Given that we did not assess the interactions with family vs. friends, or colleagues with separate items, but that these were intermingled in the frequency question, we found a less clear-cut picture than did Van Tilburg and colleagues [23], who reported that less frequent contacts with (grand-) children were associated with social loneliness, while loss of meaningful social contacts was associated with higher emotional loneliness. Yet, our findings show that an objective (as calculated across study waves in our work, rather than self-reported as in Van Tilburg et al., [23]) decrease in frequency of interactions is associated with increased social loneliness. These findings show that apart from the negative effects of lacking (or losing) specific social contacts who can offer social support in times of need, the actual frequency of interactions also contributed to how lonely a person felt during the second wave of the pandemic. That change in social contact frequency has a direct link to social loneliness is in line with theory [36], yet our study findings offer an additional insight: Of particular interest is the finding that very socially lonely individuals, when increasing their frequency of interactions, felt better at a faster rate than less socially lonely individuals, for whom social loneliness also decreased, but at a slower rate. These results underscore the need for frequent social contact to counteract feelings of loneliness, above and beyond specific close social relations; hopefully, after the end of the COVID-19 pandemic, more frequent interactions will lead to a quick reduction of social loneliness.

### Limitations

Despite offering unique information on the development of loneliness during the COVID-19 pandemic, several limitations warrant explanation. One of these is that we did not collect data before the start of the first pandemic wave of the COVID-19 in Switzerland and, therefore, we cannot compare within-person levels of loneliness before the start of the pandemic and during the second wave; still, our data clearly show longitudinal trajectories, even when considering the second wave only. Another limitation may be that given that our study period was between October and December, there could have been seasonal effects, given the association between (seasonally increased) depression and loneliness. Still, a stronger increase in emotional than social loneliness was also found in the study by Van Tilburg and colleagues [23], which compared pre-pandemic data from October and November of 2019 with data collected in May of 2020. A final limitation is that our sample is not representative of the Swiss population, as we recruited participants in a university setting, restricting the extent to which we can generalize the findings to the general public.

## Conclusions

During the second wave of the COVID-19 pandemic, individuals struggled increasingly, as they had to limit their social contacts and interactions, which led to a loss of confidants and SOS contacts and, in turn, increased feelings of emotional loneliness. Not being able to interact closely with significant others—either via compliance to the governmental recommendations or out of fear of infecting oneself or others—this unprecedented sanitary crisis made individuals more vulnerable to loneliness. It is our hope that individuals will recover quickly once the rate of vaccination in the general population increases; still, we see a risk that individuals will have difficulty restoring their social networks, as it takes time to develop social relations into confidants and SOS partners. Another problem may be that behavioral changes, including returning to a pre-pandemic level of social contacts, may not be established easily, partially because of the fear of a next COVID-19 wave, given that vaccinations may protect less efficiently from new virus mutations. Nevertheless, findings of this and other studies show that a healthy balance needs to be found between reducing interactions to reduce the spread of the virus and encouraging people to become more socially active again to counteract further mental health issues. For instance, to help lonely individuals feel closer to their loved ones, governments as well as mental health professionals should encourage the maintenance of social connections in any possible way, ideally through in-person outside contacts [21,59]. Lastly, the design of targeted interventions aimed at enhancing coping strategies especially useful to tackle the pandemic challenges may be another way to prevent mental health issues, including loneliness, from becoming a chronic risk or problem for a larger group of the population.

## Supporting information

S1 Table Multilevel models. Fixed and Random Effects of Between- and Within-Subject Covariates of Emotional and Social Loneliness.
(DOCX)
