# Supplementary material for: Loneliness during COVID-19: Development and influencing factors
Source: PLoS One. 2022 Mar 30;17(3):e0265900. doi: 10.1371/journal.pone.0265900 (PMC8967032; doi:10.1371/journal.pone.0265900)
Supplement: S1 Table — Fixed and Random Effects of Between- and Within-Subject Covariates of Emotional and Social Loneliness. (DOCX) [file pone.0265900.s001.docx]

**Appendix**

Multilevel Models with Fixed and Random Effects of Between- and Within-Subjects Covariates of Emotional and Social Loneliness (*N* = 737)

|  | Social Loneliness | | Emotional Loneliness | |
| --- | --- | --- | --- | --- |
|  | *B* | *SE* | *B* | *SE* |
| *Fixed Between-Subjects Effects* |  |  |  |  |
| Age | .0002 | .003 | -.01^**^ | .003 |
| Sex (1 = women) | .02 | .06 | -.02 | .06 |
| Married (1 = yes) | -.03 | .08 | .05 | .10 |
| Living alone (1 = yes) | -.07 | .08 | -.25^**^ | .09 |
| Subjective health | -.13^**^ | .04 | -.38^***^ | .05 |
| Education years | .03^**^ | .01 | .02 | .01 |
| Financial | -.06 | .04 | .01 | .05 |
| Student (1 = yes) | .03 | .11 | .06 | .13 |
| Employed (1 = yes) | .05 | .10 | .19 | .11 |
| Social distancing measures | .09 | .07 | .13 | .08 |
| Sanitary measures | -.12 | .10 | -.19 | .11 |
| Social media | .05^*^ | .03 | .11^**^ | .03 |
| Traditional communication | -.03 | .03 | -.06 | .04 |
| Video communication | .03 | .02 | .04 | .03 |
| N. relatives in contact | .04 | .04 | .02 | .05 |
| N. relatives as confidant | -.02 | .04 | .03 | .04 |
| N. relatives as SOS contact | -.14^**^ | .04 | -.19^***^ | .05 |
| N. friends in contact | -.01 | .04 | -.01 | .04 |
| N. friends as confidant | -.14^**^ | .05 | .06 | .06 |
| N. friends as SOS contact | -.26^***^ | .05 | -.24^***^ | .05 |
| Frequency of interactions | -.16^**^ | .04 | -.09^+^ | .05 |
| *Fixed Within-Subjects Effects* |  |  |  |  |
| Time | -.04^*^ | .02 | .03 | .02 |
| Subjective health | -.10^**^ | .03 | -.15^***^ | .03 |
| N. relatives in contact | -.04 | .02 | -.01 | .02 |
| N. relatives as confidant | -.04^+^ | .03 | -.03 | .03 |
| N. relatives as SOS contact | -.02 | .03 | -.02 | .03 |
| N. friends in contact | -.01 | .02 | -.002 | .02 |
| N. friends as confidant | .01 | .03 | -.06^*^ | .03 |
| N. friends as SOS contact | -.07^**^ | .03 | .02 | .03 |
| Frequency of interactions | -.09^**^ | .03 | -.04 | .03 |
| *Random Effects* |  |  |  |  |
| Residual variance | .22^***^ | .01 | .23^***^ | .01 |
| Intercept | .27^***^ | .02 | .39^***^ | .03 |
| Slope (frequency of interactions) | .03 | .02 | - | - |
| Slope (N. friends as confidant) | - | - | .05^*^ | .02 |
| Covariance intercept*slope | -.05^*^ | .02 | -.01 | .02 |
| -2 log likelihood (df) | 3250.78 (35) | | 3536.73 (35) | |
| AIC | 3320.78 | | 3606.73 | |
| ρ | .55 | | .63 | |
| *Notes*: N.= *Number of*. df = *degrees of freedom*. AIC = *Akaike information criterion*. ρ = *Intraclass correlation coefficient*. Unstandardized estimates and standard errors are presented. ^+^*p* < .10; ^*^*p* < .05; ^**^*p* < .01; ^***^*p* < .001. | | | | |
